# Effect of Ajwa date pits powder (*Phoenix dactylifera *L.) on body composition, lipid profile and blood pressure in patients with hyperlipidemia: A randomized clinical trial

**DOI:** 10.22038/AJP.2022.21316

**Published:** 2023

**Authors:** Parkha Riaz Nasrullah, Bismillah Sehar, Mahpara Safdar, Sadia Fatima, Falak Zeb, Jahan Shah, Atif Ali Khan Khalil, Bilal Ahmed, Ali Saad R. Alsubaie, Muhammad Haidar Zaman

**Affiliations:** 1 *Department of Human Nutrition, University of Agriculture Peshawar, KP Pakistan*; 2 *Department of Health and Social Sciences, University of Bedfordshire, UK*; 3 *Department of Environmental Design, Health and Nutritional Sciences, Faculty of Sciences, Allama Iqbal Open University Islamabad, Pakistan*; 4 *Department of Biochemistry, Khyber Medical University, Peshawar, Pakistan*; 5 *Sharjah Institute for Medical Research, University of Sharjah, United Arab Emirates*; 6 *Department of Epidemiology, School of Public Health, Nanjing Medical University, Nanjing, China*; 7 *Department of Pharmacognosy, Faculty of Pharmaceutical and Allied Health Sciences, Lahore College for Women University, Lahore, Pakistan*; 8 *Department of Pharmacology, School of Pharmacy, Nanjing Medical University, Nanjing, Jiangsu Province, PR China*; 9 *Department of Public Health, College of Public Health, Imam Abdulrahman Bin Faisal University, Dammam, Kingdom of Saudi Arabia*; 10 *Nan Shi Fu Zhong (NSFZ), Nanjing Normal University, Nanjing-China*

**Keywords:** Ajwa dates pit powder, Hyperlipidemia, Body composition, Systolic blood pressure

## Abstract

**Objective:**

To evaluate the effect of Ajwa dates pit powder (ADP) on lipid profile, body composition and blood pressure in patients with hyperlipidemia.

**Materials and Methods:**

This randomized controlled clinical study was carried out on 40 patients with total cholesterol >200 mg/dl, triglycerides >150 mg/dl and BMI >25, of either sex, aged 30-50 years, who were recruited through written consent. The patients were divided into two groups (n=20 each): the ADP and the control group (CG). All patients received the doctor’s prescribed class A statin (Rosuvastatin/ Atorvastatin) 10 mg/day, while 2.7 g ADP was given on daily basis before breakfast with lukewarm water for 40 days and the control group received the same amount of wheat flour. Body composition, blood pressure and lipid profile were determined at baseline, and after 20 and 40 days. Data were analyzed by using SPSS and GraphPad Prism.

**Results:**

ADP significantly reduced body weight (p<0.001), BMI (p<0.001), fat mass, body fat percentage, visceral fat area and waist circumference compared to the control group. Similarly, ADP significantly (p=0.000) decreased the serum level of total cholesterol and low-density lipoprotein.

**Conclusion:**

ADP may have the potential to improve dyslipidemia and obesity.

## Introduction

Globally, cardiovascular diseases (CVDs) are the most prevalent of all non-communicable diseases (NCDs) which account for an estimated 50% of deaths, out of which 31% are caused by CVDs. In Pakistan, CVDs are 19% prevalent with a 21% probability for each adult citizen to develop CVDs with the present diet and life style factors (Khan et al., 2006). Moreover, in 2013, 23.6% adults were having ischemic heart disease (Usman et al., 2014). Hyperlipidemia is a chief foundation of heart diseases. It comprises a heterogeneous group of disorders and reflects dyslipidemia characterized by an elevation in the serum total cholesterol, triglyceride (Zeb et al., 2018), and low-density lipoprotein cholesterol (LDL-C), and a noticeable decline in high-density lipoprotein cholesterol (HDL-C) concentration (Guo et al., 2014). Evidence shows that the initiation and progression of cardiovascular dysfunction including hyperlipidemia, hypercholesterolemia, and hypertension are closely related to oxidative stress (Stokes et al., 2002; Taniyama and Griendling, 2003).

Date (*Phoenix dactylifera *L.) holds an amazing prominence being the invincible provider of nutrition as well as a healing therapy (Besbes et al., 2004). Nevertheless, the present era scientists and researchers become concerned with the anti-oxidative properties, phenolic and flavonoid contents of date. These compounds have been found to counterbalance the reactive oxidative species (ROS) inside the body which prevents from metabolic processes (Aludhaib, 2015). Researches have attributed antioxidant property of date seed extract majorly due to phenolic and oleic acid contents (Ardekani et al., 2010; Amany et al., 2012). Previous studies have also stated that some phytocomponents, mainly saponins, bring out anti-hyperlipidemic action by hindering the absorption of lipids in the intestine through a resin-like action, furthermore, holding back the activity of lipase (Adeneye et al., 2010; Juárez-Rojop et al., 2012). There is some compelling evidence suggesting that flavonoids in Ajwa date seed powder have cardio-protective effects (Aludhaib, 2015). Flavonoids along with hydroxycinnamates, due to their ability of metal ion chelation and hydroxyl hydrogen ion donation prevent the LDL peroxidation in in-vitro and become the radical scavengers of ROS, and flavonoids intake is significantly and inversely connected with coronary heart disease mortality (Hertog et al., 1995).

Oleic acid (omega-9), linoleic acid (co-6), and linolenic acid (co-3) in the date seed are beneficial dietary interventions to treat hyperlipidemia by lowering LDL, and at the same time increasing HDL content in blood (Gilmore et al., 2011). Saponins in the date pits extract and showed the antihyperlipidemic effects through the suppression of pancreatic lipase (Kimura et al., 2006; Sun et al., 2012), adipogenesis inhibition (Megalli et al., 2006), and affecting the expression of appetite peptides (Kim et al., 2005). The high potassium and low sodium contents of date pits (Afiq et al., 2013), are suitable for people with hypertension (Appel et al., 1997). Additionally, the resistant starch found in the Ajwa date pit (Hamada et al., 2002), results in propionic acid production which in diet alone is responsible for inhibition of hydroxy-methylglutaryl-CoA reductase (HMG- CoA) (Bearlieu et al., 1992).

On the foundation of numerous animal research studies, as well as, considering the extraordinary nutritional worth of Ajwa date pits powder (ADP), this study possesses the manifesto of finding out the influence of the powder of Ajwa date pits upon the lipid profile, body composition and blood pressure of patients with hyperlipidemia to be considered a therapeutic remedy against heart diseases.

## Materials and Methods

**Study design and location**

A single-blind randomized controlled trial was carried out to conduct this interventional study in the Cardiac Rehabilitation Center (CRC) of Hayatabad Medical Complex, Peshawar, Khyber Pakhtunkhwa. The study was briefed to and ethical approval (IRB-23231) was granted by the Institutional Research and Ethics Board (IREB) of Postgraduate Medical Institute, Hayatabad, before the initiation of the study.

**Subjects recruitment**

A total of 40 patients visiting outpatient department (OPD) with hyperlipidemia aged 30-50 years, of either sex, were recruited. OpenEPI software was used for calculation of sample size considering alpha being 95%. A written consent form was taken from each patient prior to the enrolment. The subjects were selected randomly from Cardiology department within the Cardiac Rehabilitation Center (CRC) and they were kept blind throughout the study. 

**Inclusion and exclusion criteria**

Patients on doctor’s medication of Class-A statins (either Rosuvastatin or Atorvastatin), having hyperlipidemia with risk of CVDs, a total cholesterol >200 mg/dl (Alamgir et al., 2022), total triglycerides >150 mg/dl (Nordestgaard., 2014) and BMI >25 kg/m^2 ^were included. Pregnant and lactating women, patients with other problems such as liver, kidney or psychological diseases, physically active or athletic individuals or those having BMI below normal were excluded from the study.

**Preparation of doses**

Locally available Ajwa date pit powder (ADP) was purchased from Madinah market Saudi Arabia. The Ajwa pit powder in its bottles was stored in a dry place at room temperature. The product was then tested and packed in Pakistan Council of Scientific and Industrial Research (PCSIR) Laboratory Peshawar, after confirming the quality and taking authorization. The powder was treated with hot air at 45-50^º^C in drying oven for two hours and put in an air tight plastic bag inside a desiccator after which 2.7 g per dose per sachet was measured using an analytical balance. Food grade foil was used to make the sachets and was packed using a heat belt sealer.

**Study protocol**

The patients with hyperlipidemia (n=40) were divided into 2 groups, each group comprising 20 patients. The 1^st^ group was kept as control group (CG) receiving the prescribed statin (Rosuvastatin or Atorvastatin) 10 mg/day, while the 2^nd^ group was given 2.70 grams (2.70 g = an ample 1 teaspoon full of Ajwa date pits powder) ADP before breakfast in the morning (fasting) with 1 cup lukewarm water along with the same prescribed statin (Rosuvastatin or Atorvastatin) 10 mg/day. Assessment of body composition, blood pressure was done and blood samples were collected at baseline (day 0), and on day 20 and 40 for lipid profile analysis.

**Experiment method**

As the total study period was 40 days, the doses were started at the day of registration with the initial assessment in accordance with the inclusion criteria. For every 20 days, the patients were given a box of prepared product containing a total of 20 ADP sachets and directed to return the remaining sachets at the upcoming visit. The prescribed dose (i.e. 1 sachet) was taken by the ADP group every morning for 20 days along with the regular medication. 

**Method for blood collection**

Fasting blood was strained from the incubated vein by hospital nurse. The upper arm was wrapped with a tourniquet band to decrease the flow of blood though the veins and the puncture point was wiped with an alcohol swab. The needle was injected to draw 3-4 ml blood which was collected in tubes containing ethylenediamine tetraacetic acid (EDTA), and then shaken. These blood samples were taken to the Laboratory of Hayatabad Medical Complex for analysis. Later on, these samples were centrifuged 3000 rpm for 3-4 min at 20°C and serum was kept frozen at -80°C until analysis in polypropylene serum tubes. 

**Determination of serum lipid profile**

Serum lipid profile was determined by using enzymatic calorimetric method. Reagents were purchased from DIALAB Company, Austria and were analyzed by Microlab-300 following previous protocol (Zeb et al, 2018). The value of triglyceride was used to estimate the VLDL-c through Wilson Rule (Wilson et al., 1985). VLDL = Triglycerides (TGL)/5 (mg/dl)

**Assessment of body composition and nutritional status**

Anthropometric measurements were taken at the two time points (baseline and after 40 days) except BMI as it was measured at baseline, and on day 20 and 40. Body weight, fat mass and percent, fat-free mass, and total body water were measured using direct segmental multi-frequency bioelectrical impedance analysis (DSM-BIA; TANITA, MC-980, Tokyo/Japan). The visceral fat rating was measured by DSM-BIA machine, and the value (from 0 to 100) was converted into a visceral fat surface area by multiplying the obtained value by 10, as per the instructions of the manufacturer. Height was measured using a fixed stadiometer to the nearest 0.1 cm. BMI was calculated as Kg/m^2^ accordingly. Waist and hip circumferences were measured to the nearest 0.01 m using a non-stretchable measuring tape (Seca, Hamburg/Germany), and waist to hip ratio (WHR) was calculated according to the WHO criteria (2005).

**Blood pressure**

Using sphygmomanometer, blood pressure was noted during consultation for each patient in a relaxed seated position with their right arm entirely bare and lying upon any assistive plane. Diastolic and systolic readings were recorded twice with a time interval of 6 to 12 minutes whose mean was documented as the blood pressure (BP) of the patient.

**Statistical analysis**

Statistical Analysis was carried out using SPSS software (version 21.0) and Graphpad Prism 5. Once the data for BMI, blood pressure, cholesterol, triglyceride, HDL, LDL and VLDL was put, the results are expressed as mean±standard deviation (SD).  Repeated measure analysis was done by using ANCOVA to check within group changes, followed by Bonferroni post test. Independent T-test was applied to find out the net difference between the control and Ajwa Date pits powder-treated group and means for both tests were compared through Least Significance Difference Test at 5% level of significance.

## Results

**Figure 1:**
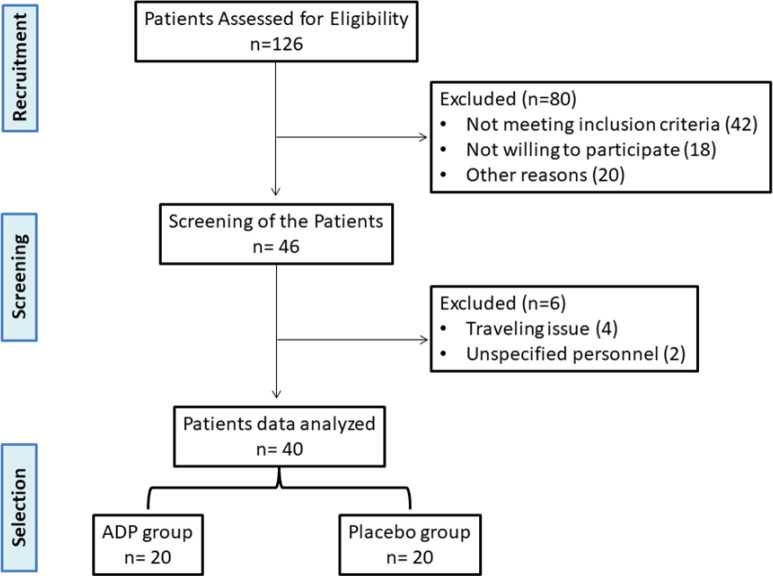
The study flow chart

In the present study, the impact of Ajwa date pits powder (ADP) was observed on body composition, blood pressure, and lipid profile among patients with hyperlipidemia. Our results demonstrated that ADP supplementation improved the nutritional status and lipid profile of the patients. A total of 126 patients were recruited. After screening, 80 patients were excluded due to not meeting the criteria and other reasons. Further 6 patients were excluded out of 46 due to travel and unspecified personnel reasons (Figure 1).

**Baseline anthropometric characteristics**

At baseline, the patients were screened and body composition and nutritional status of both groups were assessed. There was no significant difference in the body composition between the groups at baseline (Table 1).

**Table 1:** Baseline anthropometric characteristics of the study subjects

**Parameters**	**Mean±SD**		**p-value**
	**CG**	**ADP**	
Weight (kg)	89.49±12.57	89.24±13.76	NS
FM (kg)	27.52±8.74	27.10±8.27	NS
BFP (%)	30.50±8.07	30.54±8.10	NS
FFM (kg)	61.41±11.23	61.40±10.65	NS
MM (kg)	58.55±9.12	58.46±9.07	NS
TBW (kg)	44.62±8.47	44.21±3.61	NS
VFA (cm^2^)	105.10±73.02	104.23±68.04	NS
WC (cm)	95.89±14.87	95.90±18.48	NS
HC (cm)	106.21±12.45	105.92±11.87	NS
WHR	0.89±0.06	0.90±0.09	NS

BMI, Body mass index; BFP, Body fat percent; FM, Fat mass; FFM, Fat-free mass; HC, Hip circumference; MM, Muscle mass; TBW, Total body water; VFA, Visceral fat area measured by DSM-BIA; WC, Waist circumference; WHR, Waist: hip ratio

**Effect of ADP on body composition**

Further to investigate the effect of ADP consumption on body composition, we again assessed the anthropometric parameters of the patients after 40 days of ithntervention and identified that weight (p<0.001), fat mass (FM; p<0.05), body fat percentage (BFP; p<0.05), visceral fat area (VFA; p<0.05) and waist circumference (WC; p<0.05) were reduced significantly in the ADP compared to control group (Table 2). 

**Effect of ADP consumption on lipid profile**

**Figure 2:**
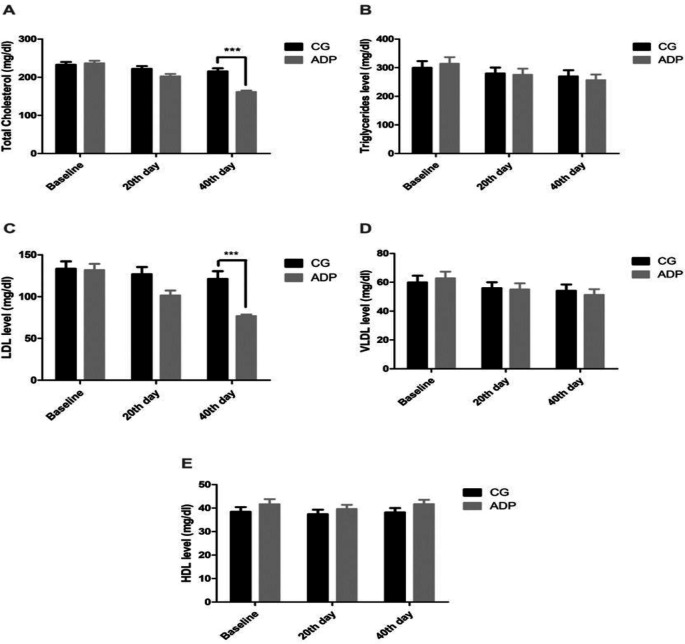
Effect of ADP consumption on lipid profile of the patients. CG= Control group, ADP= Ajwa date pits powder, LDL= Low density lipoprotein, VLDL= Very low density lipoprotein, HDL= High density lipoprotein

Moreover, we tested the serum lipid profile of the patients by using calorimetric method at three time points (i.e. baseline, and on day 20 and 40), to identify the effect of ADP on total cholesterol (TC), TG, LDL, and VLDL) and HDL (Figure 2). Intriguingly, we observed that TC (p=0.000) and LDL (p=0.000) were significantly reduced after 40 days of 2.7 g of ADF consumption compared to the control group. Although there was a reduction in the level of TG and VLDL and an increment in HDL but it was non-significant.

**Effect of ADP consumption on blood pressure and body mass index**

**Figure 3:**
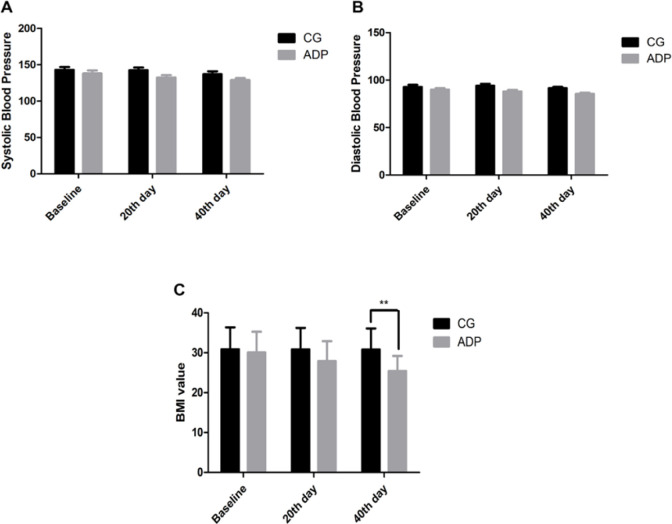
Effect of ADP consumption on blood pressure and body mass index of the patients. CG= Control group, ADP= Ajwa date pits powder, BMI= Body mass index

Further, we assessed the blood pressure and BMI of the patients, whether ADP has any effect on these parameters. There was a non-significant change observed in the mean level of systolic blood pressure (SBP) and diastolic blood pressure (DBP) while BMI was changed significantly (p=0.001) after 40 days of ADP consumption compared to the control group (Figure 3). 

**Table 2:** Effect of 40-day consumption of ADP on body composition

**Parameter**	**Mean±SD**		**p-value**
	**CG**	**ADP**	
Weight (Kg)	89.39±14.87	88.24±14.56	**
FM (kg)	27.31±9.56	26.09±9.24	*
BFP (%)	30.47±7.06	29.64±7.11	*
FFM (kg)	60.80±10.73	60.39±10.58	NS
MM (kg)	57.77±10.22	57.37±10.08	NS
TBW (kg)	43.51±7.62	43.23±7.52	NS
VFA (cm^2^)	104.96±71.17	99.14±69.02	*
WC (cm)	95.0±13.27	91.9±11.37	*
HC (cm)	105.91±10.54	104.65±10.42	NS
WHR	0.89±0.08	0.89±0.07	NS

*p<0.05, significant difference; **p<0.001, highly significant difference; Paired t-test comparing end of 40 days consumption of ADP with CG.BMI, Body mass index; BFP, Body fat percent; FM, Fat mass; FFM, Fat-free mass; HC, Hip circumference; MM, Muscle mass; TBW, Total body water; VFA, Visceral fat area measured by DSM-BIA; WC, Waist circumference; WHR, Waist: hip ratio

## Discussion

Ajwa dates and pits are previously known to scientists and researchers as a functional food, associated with numerous cellular mechanisms in lowering the elevated lipid levels, hyperglycemia, and disturbed hepatic and renal conditions *in-vitro* and in rodents. Ajwa dates (*Phoenix dactylifera *L.) fruit contains high percentage of carbohydrates, dietary fiber, fats and various types of amino acids and vitamins (Ali and Abdu, 2011; Zhang et al., 2013). They possess free-radical scavenging, antioxidant (Al-Farsi et al., 2005), antimutagenic, antimicrobial (Vayalil, 2002), hepatoprotective, nephroprotective, anticancer (Ishurd et al., 2004), and immunostimulant activities (Al-Farsi and Lee, 2008). The antioxidant and antihyperlipidemic activities are attributed to the extensive range of phenolic compounds, alkaloids, volatile oils, sterols and flavonoids being present in date pits (Baliga et al., 2011; Hong et al., 2006). 

Presently, no clinical study has been conducted to assess the effect of ADP on body composition of humans. Therefore, for the first time, we identified that 40 days of ADP supplementation with antihyperlipidemic drugs significantly decreased the body weight, fat mass, body fat percentage, visceral fat area and waist circumference of patients with hyperlipidemia. These are the first line factors associated with progression of metabolic related diseases such as hyperlipidemia, obesity, coronary artery diseases and diabetes. This significant effect on body composition and nutritional status of the patients may be due to ADP, containing nutritious and therapeutic elements. Ajwa dates are rich source of dietary fiber (Khalid et al., 2016), polyphenols (Ahmed et al., 2014), iron, calcium, potassium, phosphorous, copper, magnesium and sulfur (Hasan et al., 2010), that aid in weight loss, fat burning, decrease in level of cholesterol and glucose and beneficial effect on lipid metabolism which in turn reduces the development of cardiovascular related symptoms (Al-Turki et al., 2010; Jung et al., 2006).

In this study, ADP supplementation significantly reduced the serum level of TC and LDL while HDL increases non-significantly after 40 days. These findings indicated that ADP helps prevent the metabolic dyslipidemia by improving the risk factors in terms of body composition. However, this is the first study that investigated the effect of Ajwa dates pits powder on lipid profile of human, so we only justify our results by comparing other dates varieties. Similarly, our findings in respect of LDL, TG and HDL are supported by a previous study that used two varieties of raw dates in healthy individuals (Freha and Henchiri, 2013). The non-significant decrease and increase in serum level of TG and HDL respectively, while significant decrease in LDL supported by population based human study (Shokr et al., 2016). Consistent with our results, a study reported that Medjool date consumption by healthy subjects changed their BMI, TC, VLDL, LDL and HDL non-significantly (Rock et al., 2009).

The findings demonstrated that the blood pressure either SBP or DBP was changed non-significantly. Although previous literature showed that Ajwa dates have the potential to improve the blood pressure, our study results fail to achieve this objective probably due to low sample size and low dose of ADP. Some studies suggested that dietary intake of high potassium and low sodium has beneficial in lowering of hypertension while Ajwa dates are rich source of potassium and magnesium that play a vital role in reducing of hypertension (Ekmekcioglu et al., 2016; Assirey, 2015).

The strength of the study is that we have addressed the effect of Ajwa dates pits powder on lipid profile and blood pressure of patients with hyperlipidemia. Limitation that this study performed was not a part of any project or funded by any institution rather self-financed by the researcher. This includes the product (purchase, processing, and packing), anthropometry equipment, lab tests and logistics. In addition, due to lack of sponsorship, we were unable to provide RCT code and the human subjects were mostly reluctant to enroll, thus, a small number of subjects were included and the study was single centered.

In conclusion, the study explored the effect of Ajwa date pits powder (ADP) on body composition, lipid profile and blood pressure of patients with hyperlipidemia. The consumption of 2.7 g of ADP improved the lipid profile and body composition of the patients after 40 days. This could be a safe remedy in powder form along with other medication for the prevention of hyperlipidemia and weight loss. Therefore, researchers should conduct more studies on this valuable functional food in different domains for the prevention of metabolic diseases. 

## Conflicts of interest

The authors have declared that there is no conflict of interest.
